# RISK FACTORS ASSOCIATED WITH HEPATIC ARTERY THROMBOSIS: ANALYSIS OF 1050 LIVER TRANSPLANTS

**DOI:** 10.1590/0102-672020200004e1556

**Published:** 2021-01-25

**Authors:** Luis Eduardo Veras PINTO, Gustavo Rego COELHO, Madalena Maria Silva COUTINHO, Orlando Jorge Martins TORRES, Plinio Cunha LEAL, Ciro Bezerra VIEIRA, José Huygens Parente GARCIA

**Affiliations:** 1 Department of Surgery, Federal University of Ceará, Fortaleza, CE, Brazil; 2 Department of Surgery, Federal University of Maranhão, São Luís, MA, Brazil

**Keywords:** Risk factors, Thrombosis, Hepatic artery, Transplant, Trombose, Fatores de risco, Artéria hepática, Transplante

## Abstract

**Background:**

Hepatic artery thrombosis is an important cause of graft loss and ischemic biliary complications. The risk factors have been related to technical aspects of arterial anastomosis and non-surgical ones.

**Aim:**

To evaluate the risk factors for the development of hepatic artery thrombosis.

**Methods:**

The sample consisted of 1050 cases of liver transplant. A retrospective and cross-sectional study was carried out, and the variables studied in both donor and recipient.

**Results:**

Univariate analysis indicated that the variables related to hepatic artery thrombosis are: MELD (p=0.04) and warm time ischemia (p=0.005). In the multivariate analysis MELD=14.5 and warm ischemia time =35 min were independent risk factors for hepatic artery thrombosis. In the prevalence ratio test for analysis of the anastomosis as a variable, it was observed that patients with continuous suture had an increase in thrombosis when compared to interrupted suture.

**Conclusions:**

Prolonged warm ischemia time, calculated MELD and recipient age were independent risk factors for hepatic artery thrombosis after liver transplantation in adults. Transplanted patients with continuous suture had an increase in thrombosis when compared to interrupted suture. Re-transplantation due to hepatic artery thrombosis was associated with higher recipient mortality.

## RESUMO

**Racional:**

Trombose de artéria hepática é importante causa de falência de enxerto e complicações biliares. Fatores de risco para trombose estão relacionados aos aspectos técnicos da anastomose arterial e fatores não cirúrgicos.

**Objetivo:**

Avaliar os fatores de risco para o desenvolvimento de trombose de artéria hepática.

**Métodos:**

A amostra consta de 1050 casos de transplante hepático. Foi realizado estudo retrospectivo e transversal, e as variáveis foram avaliadas em doadores e receptores.

**Resultados:**

A análise univariada mostrou que as variáveis relacionadas a trombose de artéria hepática são: MELD e tempo de isquemia quente. Na análise multivariada, o MELD=14.5 e tempo de isquemia quente =35 min foram fatores de risco independentes para trombose de artéria hepática. No teste de prevalência para avaliação do tipo de anastomose como variável, foi observado que a sutura contínua tem maior risco de trombose quando comparada com aquela em pontos separados.

***Conclusão*:**

Tempo de isquemia quente prolongado, MELD calculado e idade do recipiente foram fatores de risco independentes para trombose de artéria hepática após transplante de fígado em adultos. Pacientes submetidos à anastomose com sutura contínua apresentaram mais trombose quando comparados com a em pontos separados. Retransplante por trombose está associado com maior mortalidade.

## INTRODUCTION

Hepatic artery thrombosis (HAT) is the most frequent and severe vascular complication of liver transplantation, being a major cause of primary dysfunction and graft loss. The incidence of this disease varies from 2-9% in adults^1^ ^,^ ^25^, and may reach 20% in some literatures^3^ ^,^ ^12^. Mortality rates range from 11-35% in adults and around 50% in pediatric transplants^22^.

The symptoms in HAT can be acute or chronic and it can be scored as early (<4 weeks) or late (>4 weeks). The most dramatic acute manifestation is fulminant ischemic hepatic necrosis, in which usually the patient rapidly develops fever, sepsis, altered mental status, hypotension and coagulopathy^23^.

Factors associated with HAT may be non-surgical and surgical. Among the non-surgical, the donor’s age (=60 years), recipients in hypercoagulable state, cases of rejection and cytomegalovirus infection are found^14^ ^,^ ^18^. Among the main surgical factors, dissection of the hepatic artery wall and technical issues in the anastomosis are found^1^.

The diagnosis of this condition is carried out by using Doppler ultrasonography as a postoperative screening and confirmed by celiac angiography or angiotomography^18^. The treatment is eminently surgical, with vast majority of patients requiring re-transplantation. In asymptomatic patients, non-surgical alternatives may be attempted, such as intra-arterial thrombolysis, with or without angioplasty, or stents^5^ ^,^ ^21^ ^,^ ^24^.

Morbidity and mortality due to early HAT, although extensively shown in the international literature when associating non-surgical factors causing HAT, have not effectively altered its incidence. The need to avoid this condition makes it necessary to assess the possibility of non-surgical factors influencing this estimate.

This study aims to analyze surgical and non-surgical risk factors of donors and recipients, associated with hepatic artery thrombosis in 1050 liver transplants in a single center and mortality after re-transplantation.

## METHODS

This work was approved by the Research Ethics Committee of the University Hospital o Walter Cantídio of the Universidade Federal do Ceará, CE, Brazil (HUWC-UFC process no. 2.438.986).

A retrospective and cross-sectional study was carried out, based on a review of medical records of 1050 patients who underwent liver transplantation per deceased donor in the Liver Transplantation Service at HUWC-UFC from May 2002 to August 2014. The inclusion criteria were all 1050 consecutive cases of liver transplantation should be carried out at this hospital. No patients were excluded from de sample.

The information obtained from the donor were: age, gender, blood type, cause of death, degree of steatosis. The recipient data: age, gender, blood type, etiology of liver disease, Model for end-stage liver disease (MELD), CHILD, calculated MELD, adjusted MELD, warm ischemia time (WIT) and cold ischemia time (CIT). 

The MELD system is based on a score that predicts severity and mortality related to end-stage liver disease. It uses serum values of total bilirubin, creatinine and INR (International Normalized Ratio). MELD sodium is a modified score that adds the value of natremia in the calculation of the prediction of mortality^10^. The Child-Turcotte-Pugh classification was created to, through the evaluation of clinical and laboratory elements, establish a score that evaluates the primary liver functions^16^. Calculated MELD is the absolute value obtained by the mathematical equation. Adjusted MELD is the value assigned to the MELD of patients with special situation. It starts with 20 points, after three months, 24 points and six months later, 29 points. The special situation was granted to patients according to Ordinance 2.600 published on October 21, 2009 by the Ministry of Health, Brazil.

The cutoff point for differentiating the anastomosis technique from the hepatic artery started from the transplant number 105; all transplants prior to this one were performed with 7-0 polypropylene continuous suture. After this number, the transplants were performed with interrupted suture using 2.5x magnification loupe and 7-0 polypropylene suture. Hepatic transplantation was performed in a universally accepted manner, divided into four stages: donor surgery, back table surgery, recipient hepatectomy and liver graft implantation^6^. We did not use aspirin or heparin of any kind for post-transplant prophilaxys.

**FIGURE 1:**
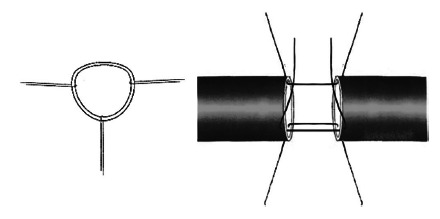
Preparation of arterial anastomosis. Passage of stay sutures for anastomosis withi nterrupted suture

Abdominal ultrasound with Doppler was carried out on the 1^st^ and 3^rd^ postoperative day. In cases of changes in Doppler or diagnostic doubt, angiotomography of the hepatic vessels was performed.

### Statistical analysis

The data were assessed by the software IBM SPSS Statistics 20 (2011). The difference of means of these same numerical variables was assessed through the parametric independent student T-test in relation to the presence or absence of thrombosis in the recipient group. Subsequently, the association of the scoring variables with respect to the donor groups was assessed through the Chi-square test of independence. The same was applied in relation to the group of recipients (there was or not thrombosis). In order to assess the risk factors, the article of the Brazilian Medical Association Guidelines^7^ was used as reference which set the following risk factors for liver donors: Age >55 years; steatosis level >30% and cold ischemia time >12 h. The other numerical variables that did not have a reference value, the ROC (Receiver Operating Characteristic) curve analysis was used to set some cut-off points for later use in multivariate logistic regression analysis. In order to assess the risk factors for the dependent variables (risk factors for thrombosis in the recipients), multivariate logistic regression was applied. The backward stepwise conditional method was used. A value of p<0.05 was considered statistically significant.

## RESULTS

The sample consisted of 1050 cases of liver transplantation from May 2002 to December 2014. Patients submitted to re-transplantation were also included in the sample. Of the total number of patients, 30 presented hepatic artery thrombosis, representing 2.8% of thrombosis in this sample.

Regarding the characteristics of donors, the majority was male (68.3%); aged between 35.8±16.1 years; blood type (ABO), types O (52.8%) and A (35.0%); degree of steatosis <30% of steatosis (74.5%); and the cause of donor death was more related to traumatic brain injury (57.3%) and stroke (33.5%, Table 1)

**TABLE 1:** Frequency distribution of the scoring variables of liver donors

Variable	n	%
Donor gender		
Male	718	68.3
Female	333	31.7
Donor age range		
< 10	16	1.5
10-19	169	16.1
20-29	259	24.6
30-39	183	17.4
40-49	180	17.1
50-59	148	14.1
60-69	75	7.1
≥ 70	19	1.8
Ignorado	3	0.3
Donor blood type		
A	368	35.0
AB	29	2.8
B	96	9.1
O	556	52.8
Ignored	3	0.3
Cause of death (n=1050)		
TBI	602	57.3
Stroke	352	33.5
FAP	17	1.6
CNS Tumor	11	1.0
Organophosphorus	10	1.0
Cerebral edema	5	0.5
Subarachnoid hemorrhage	5	0.5
Hypoxic encephalopathy	4	0.4
In blank	3	0.3
Hydrocephaly	3	0.3
Aneurysm	2	0.2
Hypoxia	4	0.4
Intoxication	2	0.2
Other causes with one case	30	2.9

TBI= trauma brain injury; FAP=firearm projectile;CNS=central nervous system

In the assessment of the recipients, there was also a greater male representation (70.4%), aged between 48.8±14.6 years, with a higher concentration ranging 50-59 years (34.4%), and the most prevalent liver diseases were cirrhosis due to hepatitis C (30.1%) and alcoholic liver cirrhosis (21.2%,Table 2).

Regarding the Child-Turcotte-Pugh score, 48.6% of the recipients were Child B, 31.3% Child C and 9.5% Child A; the calculated MELD and adjusted MELD values were, respectively, 19.6±7.0 and 21.5±8.1; and cold ischemia and warm ischemia times in the recipients were 343.1±113.6 min and 36.9±11.5 min. A significant difference (p<0.05) was found in the means of the calculated MELD and WIT variables in relation to the recipients group (with and without thrombosis, Table 3)

In the present study it was observed that only the WIT variable presented a ROC curve with a good area (65.9%), very close to the ideal (>=70%) and that was significant (p<0.05), indicating that it has good values to discriminate when donor thrombosis may or may not be present. In the other variables the ROC curve area was very low, although the calculated MELD presented significant (p<0.05). Those values were used to define cutoff points for following analysis.

**TABLE 2:** Frequency distribution of the scoring variables of liver recipients

Variable	n	%
Recipient age range		
< 10	5	0.5
10-19	58	5.5
20-29	80	7.6
30-39	92	8.7
40-49	201	19.1
50-59	361	34.3
60-69	234	22.2
≥ 70	21	2.0
Recipient blood type		
A	377	35.8
AB	37	3.5
B	117	11.1
O	521	49.5
Etiology		
HCV	317	30.1
Alcoholic cirrhosis	223	21.2
Cryptogenic	116	11.0
HBV	115	10.9
AIH	65	6.2
Fulminant hepatitis	37	3.5
Graft disfunction retx	10	1.0
PSC	17	1.6
Wilson’s disease	16	1.5
HAT	30	2.8
Buddchiari	15	1.4
Secondary biliary cirroshis	11	1.0
HCC	11	1.0
PBC	10	1.0
Other etiologies with one case	34	3.2

HCV=hepatites C vírus; HBV=hepatites B vírus;AIH=autoimune hepatitis; PSC=primary sclerosis cholangitis; HAT=hepatic artery thrombosis; HCC=hepatocarcinoma; PBC=primary biliary cirrosis

**TABLE 3:** Cutoff points of independent variables

Variable	Positive if greater than or equal to	Sensitivity	1 - Specificity
Calculated MELD	12.50	0.828	0.866
	13.50	0.759	0.829
	14.50	0.655	0.780
	15.50	0.552	0.723
	16.50	0.483	0.679
	17.50	0.483	0.631
Adjusted MELD	16.50	0.714	0.807
	17.50	0.714	0.765
	18.50	0.643	0.690
	19.50	0.536	0.651
	20.50	0.214	0.419
Warm Ischemia Time	32.50	0.724	0.585
	33.50	0.690	0.550
	34.50	0.690	0.527
	35.50	0.690	0.407
	36.50	0.655	0.391
	37.50	0.621	0.364
	38.50	0.621	0.333

**TABLE 4:** T student test: univariate analysis for presence of recipient thrombosis

Variable	Recipient thrombosis	n	Mean	SD	p
Donor age	No	1018	35.8	16.2	0.773
	Yes	31	36.6	14.7	
Recipient age	No	1021	48.8	14.6	0.369
	Yes	31	46.4	14.8	
Calculated MELD	No	982	19.7	7.0	0.043
	Yes	30	17.1	6.4	
Adjusted MELD	No	972	21.5	8.2	0.297
	Yes	29	19.9	5.4	
WIT	No	991	342.6	113.2	0.428
	Yes	31	359.0	127.6	
CIT	No	991	36.7	11.4	0.005
	Yes	31	44.0	13.4	

**FIGURE 2:**
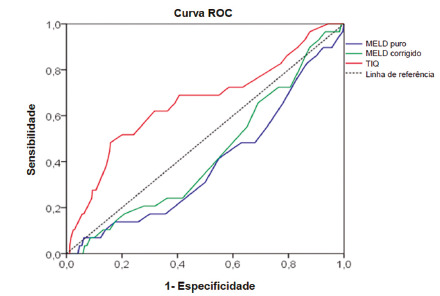
ROC curve showing cuttoff points in the independent variables (Calculated MELD, Adjusted MELD and Warm Ischemia Time)

After establishing cuttoff points, the variables were submitted to univariate (Table 4) and multivariate analysis (Table 5).

After 17 stages of selection by the backward stepwise conditional method, only warm ischemia time variables (=35), calculated MELD (=14.5) and recipient age (=42 years) were significant for thrombosis in liver recipients - all of them risk factors for thrombosis (OR>1).

**TABLE 5:** Multivariate logistic regression of the recipient thrombosis

	Variable in the equation	p	OR	IC95% OR	
				Lower	Upper
Step 1a	Recipient gender (male)	0.183	0.57	0.25	1.30
	Recipient age (=42 years)	0.001	4.96	2.26	10.93
	Recipient age (=55 years)	0.004	6.29	1.78	22.20
	Recipient blood type (A, AB or B)	0.863	0.93	0.41	2.11
	Calculated MELD (= 14.5)	0.034	2.35	1.07	5.18
	Adjusted MELD(= 19)	0.374	0.67	.273	1.629
	Cold ischemia time(=5 h)	0.290	0.63	.266	1.485
	Warm ischemia time (=35)	0.008	3.85	1.43	10.38
	Constant	0.363	0.27		

The prevalence of thrombosis in patients with continuous suture was 6.7%; by switching to interrupted suture this prevalence fell to 2.5%. Interrupted suture significantly reduced the likelihood of thrombosis (Table 3). Patients with continuous suture had an increase in thrombosis when compared to interrupted suture. When the prevalence ratio test was carried out, it was observed that in transplants recipients with thrombosis there was a higher death rate.

**TABLE 6:** Association of anastomosis type and thrombosis in liver transplant recipients

Recipient thrombosis	Anastomosis			Total	RP	p	
	Continuous suture	%	Interrupted suture	%			
Yes	7	6.7	24	2.5	31	2.63	0.018
No	98	93.3	923	97.5	1021		
Total	105	100.0	947	100.0	1052		

## DISCUSSION

Despite its low incidence, hepatic artery thrombosis is usually a devastating issue that requires re-transplantation and is associated with significant morbidity and mortality^25^. In this study, prolonged WIT, calculated MELD and recipient age were independent risk factors for HAT after liver transplantation in adults.

Piscaglia *et al*.^19^ in a study with 255 patients, presented via logistic regression the age >60 as a risk factor for HAT (OR for age >60 years; p=0.017). In addition, Marudanayagan *et al*.^15^ also showed that MELD =23 and age =55 years are associated with a better outcome after liver transplantation.

Despite the scarce literature showing the recipient age as a risk factor, this study revealed an influence of age >42 years as an independent risk factor for thrombosis. It is suggested that this fact is probably associated with a higher risk of systemic arterial disease (atherosclerosis) and increased comorbidities that are more common in patients with greater age.

MELD is a variable highly assessed but has not been linked to the risk of HAT directly and is usually related to graft loss and increased morbidity and mortality of patients. Grat *et al*.^11^ showed in a study of 786 recipients that high MELD is an independent risk factor for lower graft survival and may indirectly contribute to late HAT. Dudek *et al*.^9^ also showed lower graft survival in patients with high MELD.

In this study, calculated MELD was an independent risk factor for hepatic artery thrombosis, and although not directly associated with HAT, some publications seem to confirm the findings. Bonney *et al*.^4^ showed in 1090 transplants performed that MELD >30 associated with a high donor risk index (DRI) increased 2-fold the risk of vascular complications when compared to low-DRI donors. This probable relation identified in the study may be related to a greater severity of the recipient cirrhosis, since a higher MELD is directly associated with the degree of recipient worsening clinical condition, therefore with a greater risk of graft dysfunction, increased arterial resistance, and secondary hepatic artery thrombosis.

In this series, the cold ischemia time showed no relation to HAT; on the other hand, the warm ischemia time was a risk factor for thrombosis in the univariate analysis and confirmed in the multivariate analysis. The literature has already shown that increased surgical time, prolonged cold ischemia time and prolonged warm ischemia increase the risk of early HAT.

Although often cited, WIT is not extensively evaluated and to our knowledge, there are no publications showing it as a risk factor. The WIT average showed in this study was 36.5 min, which may be associated with intercurrences that increase this time intraoperatively, such as portal vein thrombosis not previously identified or the need of hemostasis of caval anastomosis bleeding, both conditions that could increase WIT.

In this study, surgical factors related to HAT were also assessed. The first transplants performed, more specifically from 1 to 105, were carried out by using continuous suture using 7-0 or 8-0 polypropylene wire. This type of anastomosis presented a prevalence of 6.5% of hepatic artery thrombosis. Zhao *et al.* ^29^, in 72 consecutive cases of liver transplantation using a microvascular surgery technique, with arterial interrupted suture and using a 3.5x loupe, presented only 1.4% of HAT. From the transplant number 106 to 1050, we opted for a technical modification in the arterial anastomosis. The interrupted suture was performed by using 7-0 or 8-0 polypropylene surgical thread, using loupes between 2.5x and 4.0x, based on the preference of the main surgeon and first assistant.

Starzl *et al.* ^26^ inferred in their publication, over 25 years ago, the importance of meticulous arterial reconstruction and the use of microsurgical techniques. Mori *et al.* ^17^ introduced the concept of microsurgery for reconstruction of hepatic artery anastomosis; their publication emphasizes the use of the microvascular technique with the advent of microscopes or loupes and the use of interrupted suture showing superiority over the conventional technique.

**FIGURE 3:**
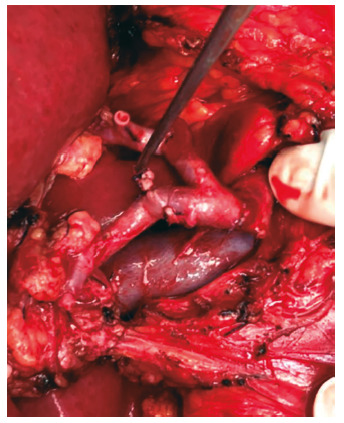
Final aspect of the hepatic artery anastomosis with interrupted suture

Arterial anastomoses in this service are preferably performed after widening the artery extremity to increase its diameter. The preparation of the arterial anastomosis is painstaking, avoiding direct clamping of the arterial wall, delicately handling it. The anastomosis is maintained as rectified as possible in order to prevent kinking (Figure 3).

The importance of increasing the arteries diameter and the use of loupes was demonstrated in the study by Li *et al.* ^13^, with a sample of 187 recipients in interventricular transplants, increasing the arteries diameter, which were on average 2.5 mm, which doubled in size by obliquely sectioning them; also showed that the use of loupe with 4.5x magnification presents results similar to the use of the microscope.

In this sample, when comparing the types of anastomosis, it was observed an incidence of thrombosis of 2.5% in patients with interrupted suture. It was concluded that recipients who have anastomosis in continuous suture have 263% more thrombosis when compared to interrupted suture.

Albeit other surgical variables were not addressed in the present study, local and technical factors in the anastomosis have great influence on the results. Tzeng, Hsieh, and Chen^27^ have published a minor study showing benefits of interrupted suture in cases of arterial wall dissection, increasing surgical time in only 20 min when compared to continuous suture*.* Zheng *et al.* ^30^, in a publication with 198 patients, compared interrupted suture with continuous suture, showing an incidence of HAT at 1% and 6.3%, respectively. Rela *et al.* ^20^ in a published technical modification similar to the one used in this service, showed an incidence of only 1.3%. In our study, even in cases of anatomical variation with arterial reconstruction and vascular grafts, anastomosis with the arterial stump of the aortohepatic graft was performed at separate points, not considering these factors.

We also analyzed the rate of re-transplantation for thrombosis and the mortality in cases of re-transplantation. Regarding the re-transplantation rate for thrombosis, all HAT, whether early or late, were treated at re-transplantation. Of the patients re-transplanted via HAT, 40% died, showing that in transplants recipients with thrombosis there was a higher rate, with a 559% increase in mortality. Liver transplantation is also associated with the emergence of sarcopenic obesity; however, similarly to the study by Anastácio et al.^2^ this relationship was not observed in our study.

As already reported by Stange *et al.* ^25^ and Oh *et al.* ^18^, regardless of the measures performed to prevent hepatic artery thrombosis with the use of anticoagulants or antiplatelet drugs, the literature shows a mortality of early HAT around 11-56%, and the rate of re-transplantation can reach up to 83%. It can be justified that our high rate of re-transplantation is due to the low accessibility and experience of the interventional radiology/vascular surgery team in endovascular procedures, which could benefit patients with non-surgical HAT. Non-surgical risk factors suggest better prevention or screening to try decreasing the risk of thrombosis in patients with the variables found.

Thus, the interrupted suture seems to be superior from the technical standpoint, as it reduces the risk of local complications, there is a greater accuracy at each suture and with the use of microsurgery techniques there is a greater care with the handling of the artery causing less injury and dissection of the arterial wall. However, the literature shows that other technical variables, such as number of anastomoses, anatomical variation and complex reconstructions, are risk factors for HAT, requiring technical and randomized studies, in order to compare the influence of the surgical technique on the HAT development.

## CONCLUSION

Prolonged warm ischemia time, calculated MELD and recipient age were independent risk factors for HAT after liver transplantation in adults. Interrupted suture significantly reduced the likelihood of thrombosis. Transplanted patients with continuous suture had an increase in thrombosis when compared to interrupted suture. Re-transplantation due to hepatic artery thrombosis was associated with higher recipient mortality.
